# Reducing Greenhouse Gas Emissions and Modifying Nitrous Oxide Delivery at Stanford: Observational, Pilot Intervention Study

**DOI:** 10.2196/64921

**Published:** 2025-01-09

**Authors:** Eric P Kraybill, David Chen, Saadat Khan, Praveen Kalra

**Affiliations:** 1 Stanford Hospital Stanford, CA United States

**Keywords:** anesthetic gases, emissions, green house gas, sustainability, pilot study, electronic health record, implementation, nitrous oxide, global warming

## Abstract

**Background:**

Inhalational anesthetic agents are a major source of potent greenhouse gases in the medical sector, and reducing their emissions is a readily addressable goal. Nitrous oxide (N_2_O) has a long environmental half-life relative to carbon dioxide combined with a low clinical potency, leading to relatively large amounts of N_2_O being stored in cryogenic tanks and H cylinders for use, increasing the chance of pollution through leaks. Building on previous findings, Stanford Health Care’s (SHC’s) N_2_O emissions were analyzed at 2 campuses and targeted for waste reduction as a precursor to system-wide reductions.

**Objective:**

We aimed to determine the extent of N_2_O pollution at SHC and subsequently whether using E-cylinders for N_2_O storage and delivery at the point of care in SHC’s ambulatory surgery centers could reduce system-wide emissions.

**Methods:**

In phase 1, total SHC (Palo Alto, California) N_2_O purchase data for calendar year 2022 were collected and compared (volume and cost) to total Palo Alto clinical delivery data using Epic electronic health records. In phase 2, a pilot study was conducted in the 8 operating rooms of SHC campus A (Redwood City). The central N_2_O pipelines were disconnected, and E-cylinders were used in each operating room. E-cylinders were weighed before and after use on a weekly basis for comparison to Epic N_2_O delivery data over a 5-week period. In phase 3, after successful implementation, the same methodology was applied to campus B, one of 3 facilities in Palo Alto.

**Results:**

In phase 1, total N_2_O purchased in 2022 was 8,217,449 L (33,201.8 lbs) at a total cost of US $63,298. Of this, only 780,882.2 L (9.5%) of N_2_O was delivered to patients, with 7,436,566.8 L (90.5%) or US $57,285 worth lost or wasted. In phase 2, the total mass of N_2_O use from E-cylinders was 7.4 lbs (1 lb N_2_O=247.5 L) or 1831.5 L at campus A. Epic data showed that the total N_2_O volume delivered was 1839.3 L (7.4 lbs). In phase 3, the total mass of N_2_O use from E-cylinders was 10.4 lbs or 2574 L at campus B (confirming reliability within error propagation margins). Epic data showed that the total N_2_O volume delivered was 2840.3 L (11.5 lbs). Over phases 2 and 3, total use for campuses A and B was less than the volume of 3 E-cylinders (1 E-cylinder=1590 L).

**Conclusions:**

Converting N_2_O delivery from centralized storage to point-of-care E-cylinders dramatically reduced waste and expense with no detriment to patient care. Our results provide strong evidence for analyzing N_2_O storage in health care systems that rely on centralized storage, and consideration of E-cylinder implementation to reduce emissions. The reduction in N_2_O waste will help meet SHC’s goal of reducing scope 1 and 2 emissions by 50% before 2030.

## Introduction

Reducing greenhouse gas (GHG) emissions is a priority that must be addressed to reduce climate change and its negative impacts on earth and its inhabitants. The US Environmental Protection Agency (EPA) classifies GHG emissions into different categories, with scope 1 emissions defined as direct GHG emissions from sources that are controlled by organizations, including health care systems, and scope 2 emissions being indirect GHG emissions associated with the purchase of electricity, heat, steam, or cooling [1]. Stanford Health Care (SHC) has signed on to the US Department of Health and Human Services’ pledge to reduce its scope 1 and 2 emissions by 50% by 2030 [2]. Within the medical sector, inhalational anesthetic gases that are directly released into the atmosphere are a major source of potent GHGs. Thus, there is a fertile opportunity to reduce GHGs by reducing the emission of anesthetic gases [3]. By collecting annual emissions data within the SHC system, improvements to sustainability and infrastructure could be explored.

Global warming potential (GWP) represents the energy a gas is able to absorb relative to carbon dioxide (CO_2_), with a larger GWP representing increased planetary warming [4]. The environmental impacts of 2 inhaled anesthetic gases over a 100-year period (ie, global warming potential of GHGs over a 100-year period [GWP100]) are particularly relevant: desflurane, a volatile halogenated agent with particularly high GWP100 of 2540, and nitrous oxide (N_2_O) with a lower GWP100 of 298 but used in much higher volumes than other anesthetic gases, and with longer half-life compared to CO_2_, leading to lasting environmental consequences [5]. Further, because of its low clinical potency, large amounts of N_2_O must be stored for use, increasing the chance of pollution through leaks. Centrally piped cryogenic liquid, centrally piped gas, and portable E-cylinders are the standard options for delivering N_2_O [6]. Miles of pipes and innumerable valves in centrally piped systems lead to an abundance of leaks, contributing to excessive loss and waste [6]. While desflurane has already been discontinued from routine clinical use at SHC, we aimed to determine the degree to which N_2_O emissions could be reduced and waste prevented, building on prior studies highlighting the waste of N_2_O in other institutions [7].

## Methods

### Phase 1

To begin investigating N_2_O emissions, purchase data (volume and cost) were collected and compared to total use data (clinical delivery) using the Epic SlicerDicer tool, part of the Epic electronic health record (EHR) [8]. Epic yearly clinical use data for N_2_O are available per clinical service in the SHC’s operating rooms. Gas losses in the system can be estimated by comparing documented gas delivery at the point of care with the volume of N_2_O purchased. Initial data analysis revealed a drastic amount of lost N_2_O, leading us to perform a pilot study (phase 2, E-cylinder implementation) to enable remediation aimed at reducing N_2_O emissions.

### Phase 2

Using the Institute for Healthcare Improvement framework of “Plan, Do, Study, Act” for performance improvement [9], a pilot study was conducted in the 8 operating rooms of the SHC campus in Redwood City, California (campus A). E-cylinder canisters were deployed in each operating room and all central N_2_O pipelines were disconnected. EHR documentation of gas delivered in liters (volume) was compared to measured E-cylinder mass. To verify use and track N_2_O leaving each tank, the E-cylinders were weighed before and after use on a weekly basis with the difference in mass converted to volume (liters). Since the measured pressure remains the same as long as liquid remains in the cylinders, pressure differences cannot be used for measuring N_2_O flow until only gas is left (at which point the pressure drop correlates with the amount of gas being removed) [10]. By using the conversion of 1 lb (0.45 kg) of N_2_O being equal to 247.5 L [6], the volume of N_2_O dispensed could be calculated. Total calculated volume leaving the E-cylinders based on measured mass was compared to total volume delivered according to Epic data.

### Phase 3

Following the results of phase 2, a secondary study was conducted in 16 operating rooms at Blake Wilbur Drive Palo Alto, California (campus B). Phase 3 used the same methodology as phase 2 over a 3-week period.

### Ethical Considerations

Due to the nature of the research and institutional approval, no IRB approval was necessary. No identifying patient data was used as we only measured nitrous oxide gas delivery and utilization.

## Results

### Phase 1

According to the Stanford Medicine Sustainability Program Office [2], the annual Palo Alto SHC 2022 Scope 1 emissions were 19,374 MTCO_2_e (metric ton of CO_2_ equivalent, the standard unit for comparing different GHGs to quantify their environmental impact and GWP) of which medical gases (including N_2_O, CO_2_, sevoflurane, and isoflurane) represented 4862 MTCO_2_e. N_2_O contributed 4590 MTCO_2_e of the medical gases. Thus, medical gases account for 25.1% of all SHC scope 1 emissions, and N_2_O alone accounts for 94.4% of those emissions (or 23.7% of the total).

Annual clinical usage of N_2_O in 2022 per Epic data (Table 1) was 780,882.2 L (3155.1 lbs or 1431.1 kg), with the greatest use being for orthopedic surgery, general surgery, and neurosurgery cases. However, the total amount of N_2_O purchased was 8,217,449 L (33,201.8 lbs or 15,060.1 kg), at a total cost of US $63,298. Thus, only 9.5% of the total purchased N_2_O was actually delivered to patients, and 90.5% (or US $57,285 worth) was wasted.

**Table 1:** Annualized data comparing centralized N_2_O system to hypothetical E-cylinders for Stanford Health Care (SHC; all campuses).

	Amount purchased (L)	Cost (US $)	Amount used (L)	Amount lost as waste (L)
Centralized system	8,217,449	63,298	780,882.2	7,436,566.8
E-cylinders	780,882.2^a^	6015	780,882.2	0^b^

^a^Amount needed to purchase with zero surplus based on use data under experimental conditions.

^b^Annualized E-cylinder data are extrapolated from experimental conditions; real-world conditions may vary.

With these data indicating a loss of greater than 90% between storage tanks and clinical use, a highly inefficient storage and pipeline system was recognized. The proposed solution (for phase 2 of the study) was to decommission the storage tanks and pipelines and switch to portable E-cylinders that stored and delivered N_2_O at the point of care.

### Phase 2

The change in mass of the E-cylinders indicated that N_2_O use at campus A totaled 7.4 lbs (3.4 kg), or a volume of 1831.5 L, over the 5-week study period. Epic data showed total N_2_O volume delivered to be 1839.3 L calculated to 7.4 lbs (3.4 kg; consistent with the measured 7.4 lbs). Using the standard of 1 E-cylinder=1590 L or 6.4 lbs (2.9 kg) [11], total use equaled 1.16 E-cylinders.

### Phase 3

The E-cylinder change in mass indicated that N_2_O use at campus B totaled 10.4 lbs (4.7 kg), or 2574 L, over the 3-week data collection period. Epic data showed total N_2_O volume delivered to be 2840.3 L calculated to 11.5 lbs (5.2 kg; compared to the measured 10.4 lbs, which would be equivalent to 1.63 E-cylinders) [11].

## Discussion

### Principal Findings

Results from phase 1 corroborate findings from previous studies in the United Kingdom and Portland, Oregon [12,13], which reveal excessive waste from centralized storage of N_2_O and pipe systems for delivery. Phases 2 and 3 of this study, from 2 different SHC campuses, demonstrate the efficient, cost-effective elimination of waste through substitution of E-cylinders with storage and delivery at the point of care. In phases 2 and 3, avoidable N_2_O emissions were almost completely eliminated (Multimedia Appendix 1). The discrepancy between actual weighed N_2_O and Epic-reported use for campus A was 7.8 L, or <0.1 lb (<0.1 kg). Campus B had a greater discrepancy with the difference in actual weighed N_2_O and Epic-reported use being 266.3 L, or 1.1 lbs (0.45 kg). The amount of gas delivered according to the EHR was greater than the amount actually measured at the source, potentially accounted for by limited precision of the scales used to weigh the E-cylinders (only to 0.1-lb increments), or accidental reconnection of N_2_O pipelines in one operating room during phase 3. This issue was detected after 1 week and immediately rectified.

E-cylinders provide an efficient and effective solution, but they hold limitations. E-cylinders must be stored properly to ensure that they do not present a catalyst in the event of a fire [14]. However, no policy implementation is required as E-cylinders are already in use in operating rooms and costs associated with storage can be offset by the N_2_O saved. Ready accessibility, lower cost, reduced supply chain issues, and efficiency of E-cylinders far outweigh the abovementioned disadvantages.

### Limitations

The limitations of this study include the fact that real-world use and waste may vary from our experimental conditions, likely incurring greater losses. If e-cylinder valves are accidentally left open, losses may simulate those from centralized pipelines until the valve is closed [6] or the E-cylinder is emptied. The amount of N_2_O to be purchased would need to be greater than the amount used in our example (Table 1), to provide surplus in the E-cylinders as well as spare E-cylinders. Prospective estimates of volume when making a purchase order would likely exceed actual use. Both recording and documentation of N_2_O readings and the scale measurements are susceptible to error.

### Conclusions

Converting the delivery of N_2_O from centralized storage to point-of-care E-cylinders has dramatically reduced waste and expense with no detriment to patient care. Stanford’s pledge to reduce scope 1 and 2 emissions by 50% can be achieved and even surpassed if this practice is changed in all SHC locations. The introduction of E-cylinders will provide a nondisruptive means for immediately decreasing emissions while continuing to provide optimal anesthetic care. Pilot studies throughout Stanford’s campuses continue, with the goal of removing the centralized N_2_O system and switching to E-cylinders at other sites, thereby significantly reducing anesthetic GHG emissions. Efforts to reduce GHG emissions may begin locally but have applications globally. Reducing the anesthetic carbon footprint of health care organizations is necessary for our planet and can begin with the reduction of wasteful emissions.

## Appendix

Multimedia Appendix 1 Reduction in N_2_O emissions per metric ton of CO_2_ equivalents by switching from the original central supply to portable supply E-cylinder storage.

## Abbreviations

EHR: electronic health record
EPA: Environmental Protection Agency
GHG: greenhouse gas
GWP: global warming potential
GWP100: global warming potential of GHGs over a 100-year period
MTCO_2_e: metric ton of carbon dioxide equivalent
N_2_O: nitrous oxide
SHC: Stanford Health Care
