# Supplementary figures and images for: Reducing Greenhouse Gas Emissions and Modifying Nitrous Oxide Delivery at Stanford: Observational, Pilot Intervention Study
Source: JMIR Perioper Med. 2025 Jan 9;8:e64921. doi: 10.2196/64921 (PMC11757946; doi:10.2196/64921)

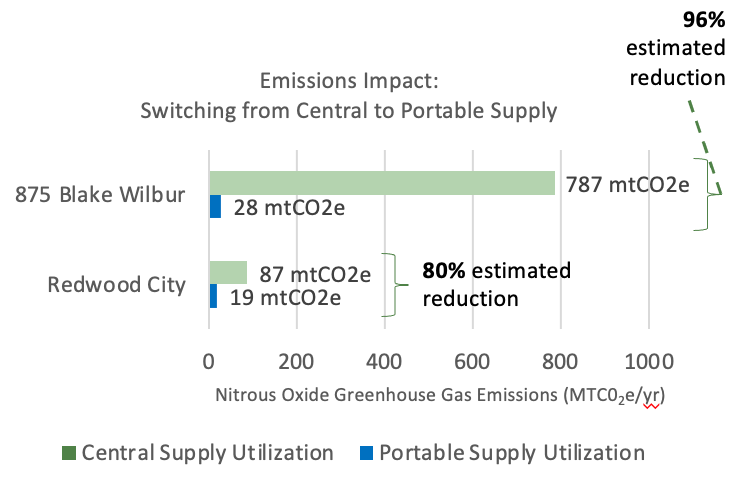

Supplement: Multimedia Appendix 1 [file periop_v8i1e64921_app1.png]
